# Surveillance of hospital acquired infections according to ECDC definitions in Polish hospital - a pilot study

**DOI:** 10.1186/2047-2994-4-S1-P276

**Published:** 2015-06-16

**Authors:** A Różańska, J Wójkowska-Mach, M Bulanda

**Affiliations:** 1 Chair of Microbiology, Jagiellonian University Medical College, Kraków, Poland

## Introduction

Hospital acquired infections (HAIs) registration is one of the essential elements of the infection control programs. In 2011 Polish Society of Hospital Infections has developed HAIs surveillance program convergent with current recommendations in the European Union based on the ECDC HAI-Net system.

## Objectives

The aim of this work was to analyse epidemiological situation in six intensive care units from the hospitals taking part in the surveillance system based on ECDC HAI-Net recommandations.

## Methods

This work presents the results of HAIs registration in the intensive care units, collected on the basis of guidelines and criteria for infections’ identification according to HAI-Net recommendations. Twenty one hospitals, mainly from the southern Poland, participated in the program since 2012. Presented data were gathered in six intensive care units, reporting cases of hospital acquired pneumonia (PN), bloodstream infections (BSIs) and urinary tract infections (UTIs), in 2013 and 2014. Continuous, active surveillance method was used. HAIs cases were detected by infection control teams.

## Results

Epidemiological situation in the studied wards was diverse – HAI cases were reported in between 6.25% - 26.64% patients hospitalized in these units (for 100 admissions). The highest incidence rates were observed for PN (max. 19.47%). The incidence rates for all forms of infections varied in the broad range between the studied wards: for PN: 0.76%>19.47%, primary BSI: 1.10% - 16.81%, secondary BSI: 0.37% - 7.25% and for UTI: 0.0% - 13.27%.

The most often identified forms of PN were PN-4 and PN-5. Only in two ICUs hospital acquired pneumonia cases were microbiologically confirmed according to PN-1 definition criteria.

## Conclusion

Presented results point at significant differences in epidemiological situation in individual units. There were also important differences in the routinely used microbiological methods of PN confirmation.

Introducing HAI-Net recommendations concerning HAIs surviellance in Polish hospitals encounters difficulties connecting with the lack of principles and requirements on the national level.

## Disclosure of interest

None declared.

